# The Equine Reproductive Microbiota: Composition, Dynamics, Dysbiosis, and Implications for Fertility in Mares and Stallions

**DOI:** 10.3390/ani16091414

**Published:** 2026-05-05

**Authors:** Urtė Pelenė, Artūras Šiukščius, Rasa Nainienė, Inga Merkelytė, Rūta Šveistienė

**Affiliations:** Department of Animal Breeding and Reproduction, Animal Science Institute, Lithuanian University of Health Sciences, R. Zebenkos 12, 82317 Baisogala, Lithuania; arturas.siukscius@lsmu.lt (A.Š.); rasa.nainiene@lsmu.lt (R.N.); inga.merkelyte@lsmu.lt (I.M.); ruta.sveistiene@lsmu.lt (R.Š.)

**Keywords:** equine reproductive microbiota, uterine microbiota, vaginal microbiota, seminal microbiota, dysbiosis, endometritis, fertility, 16S rRNA sequencing

## Abstract

The equine reproductive microbiota has emerged as an important factor influencing reproductive health and fertility in both mares and stallions. Traditionally, the equine uterus was considered sterile, and microbial presence was interpreted primarily in the context of infection. However, sequencing-based studies have demonstrated that the reproductive tract, including the uterus and semen, contains detectable microbial communities or microbial DNA signatures, challenging this traditional paradigm. In mares, the vaginal microbiota is consistently dominated by *Firmicutes*, *Bacteroidetes*, *Proteobacteria*, and *Actinobacteria*, whereas the uterine environment represents a low-biomass niche in which interpretation is complicated by contamination risk and the inability of sequencing-based methods to distinguish viable from nonviable microorganisms. Culture-based studies consistently identify opportunistic pathogens such as *Streptococcus equi* subsp. *zooepidemicus*, *Escherichia coli*, and *Pseudomonas aeruginosa* in association with endometritis and persistent breeding-induced endometritis, while sequencing-based studies suggest broader community-level dysbiosis rather than simple pathogen presence. In stallions, semen is not sterile and commonly contains taxa such as *Porphyromonas*, *Corynebacterium*, *Peptoniphilus*, and other opportunistic bacteria that may influence sperm quality and microbial transmission to mares during breeding. However, most reported associations remain correlative, and direct longitudinal evidence for persistent stallion-to-mare microbial transmission is limited. This review synthesizes current evidence on microbial composition, hormonal influences, dysbiosis, and reproductive implications of the equine reproductive microbiota, integrating culture-based and sequencing-based findings while emphasizing methodological limitations associated with low-biomass samples. Improved understanding of these microbial ecosystems may support more evidence-based reproductive diagnostics and microbiome-informed fertility management in horses.

## Simple Summary

Microorganisms inhabiting the reproductive tract may influence fertility in both mares and stallions. Traditionally, the equine uterus was considered sterile, and microbial detection was interpreted primarily as evidence of infection. However, recent sequencing-based studies have shown that bacterial DNA is also present in clinically healthy reproductive tissues, suggesting a more complex microbial environment. In mares, changes in microbial balance have been associated with endometritis, persistent breeding-induced endometritis, and reduced fertility. In stallions, semen contains diverse bacterial communities that may affect sperm quality and serve as a source of microbial transfer during breeding. Interpreting these findings remains challenging because culture-based and sequencing-based methods provide different types of information, and uterine samples are particularly prone to contamination due to low microbial biomass. This review summarizes current knowledge of the equine reproductive microbiota in mares and stallions, focusing on microbial composition, dysbiosis, reproductive disorders, and clinical implications. A better understanding of these microbial ecosystems may improve reproductive diagnostics, support more rational antimicrobial use, and contribute to more effective fertility management in horses.

## 1. Introduction

Equine reproductive performance is inherently variable, with conception rates, pregnancy outcomes, and age-related subfertility reducing breeding efficiency in both mares and stallions [1,2]. Early embryonic loss and age-associated decline in reproductive performance are well-documented limitations to fertility in mares [3,4], while in stallions, subfertility may result from congenital, acquired, or management-related factors affecting semen quality, reproductive tract health, and breeding performance [5,6,7]. Population-level studies also demonstrate variability in reproductive outcomes across breeds and management systems [8,9,10,11]. Collectively, these limitations highlight the need for a more integrated understanding of the biological processes influencing fertility in horses [1,2].

Historically, the equine uterus was considered a sterile environment, and microbial detection was interpreted primarily as evidence of infection [12,13,14]. This view was shaped by culture-based methodologies, which detects only viable microorganisms capable of growth under laboratory conditions and therefore provide a limited perspective on the reproductive microbial environment [13,14]. Nevertheless, culture-based diagnostics remain clinically important because they enable detection of viable pathogens associated with endometritis, including *Escherichia coli* and *Streptococcus equi* subsp. *zooepidemicus* [12,13,14].

The introduction of culture-independent, sequencing-based approaches has substantially challenged this traditional view. Recent equine studies have reported bacterial DNA signatures within the reproductive tract, including the uterus, suggesting that the equine reproductive environment may not be strictly sterile [15,16,17,18]. For example, Thomson et al. found that uterine samples from clinically healthy mares contained bacterial DNA dominated by *Proteobacteria*, *Firmicutes*, and *Bacteroidetes* [17]. Similarly, Holyoak et al. identified a diverse uterine microbial signal and proposed the existence of a core microbiome shared across mares, although its composition varied with geographical location [16]. However, these findings should not be interpreted uncritically as evidence of stable colonization or a functionally active resident microbiota.

Findings from culture-based and sequencing-based methods are not directly comparable and must be interpreted within their methodological context [15,19,20]. Culture-based approaches identify viable organisms capable of growth under defined laboratory conditions, while sequencing-based methods detect total microbial DNA, including DNA from nonviable cells and extracellular material [19,20]. As a result, sequencing typically reveals broader taxonomic diversity than culture, but it does not establish microbial viability or ecological function [19,20]. This methodological distinction is central to interpreting reproductive microbiome data and explains many apparent discrepancies across studies.

Interpretation of sequencing-based findings in the equine uterus is further complicated by the low microbial biomass of uterine samples, which increases susceptibility to contamination from reagents, instruments, and the environment [15,20,21,22,23,24,25,26]. In low-biomass settings, even small amounts of contaminant DNA can disproportionately influence sequencing results and lead to misinterpretation of microbial composition [21,22,23,24,25,26]. Consistent with this concern, equine uterine microbiome studies have acknowledged that some detected taxa may reflect contamination rather than biologically relevant uterine microorganisms [16,17]. Accordingly, uterine sequencing data in mares requires particularly cautious interpretation.

Despite these limitations, accumulating evidence suggests that reproductive tract microorganisms may be relevant to reproductive physiology and fertility. In mares, alterations in microbial composition have been associated with endometritis and reduced reproductive performance, particularly in persistent breeding-induced endometritis (PBIE) [12,27,28]. Sequencing-based studies further suggest that microbial community structure may interact with uterine immune responses and clearance mechanisms, although current evidence remains largely observational [16,17,18,29]. Thus, causal relationships between microbial patterns and reproductive pathology remain poorly defined, and many proposed mechanisms are still extrapolated from broader microbiome literature rather than demonstrated directly in horses [30,31].

In addition to the mare, the stallion reproductive tract has increasingly been recognized as a source of reproductive microbial exposure. Sequencing-based studies show that stallion semen is not sterile and contains diverse bacterial communities [32,33,34,35,36]. These microorganisms may influence sperm quality and contribute to microbial transfer during natural mating or artificial insemination; however, direct longitudinal evidence demonstrating sustained transmission or colonization in horses remains limited [32,33,34,35,36,37].

Taken together, current evidence indicates that both mares and stallions harbor detectable microbial signals within their reproductive tracts, but the biological significance, stability, and functional activity of these microorganisms remain incompletely understood.

The aim of this review is to synthesize current evidence on the composition, dynamics, and clinical relevance of the equine reproductive microbiota in mares and stallions. By critically integrating findings from culture-based and sequencing-based studies with reproductive physiology and pathology, this review provides a framework for interpreting microbiota data in equine reproductive medicine and identifies key priorities for future research.

## 2. Materials and Methods

This review was conducted as a narrative synthesis aimed at integrating current evidence on microbial communities inhabiting the reproductive tract of mares and stallions, with a particular focus on microbial composition, hormonal influences, host–microbe interactions, dysbiosis, and clinical implications for fertility. Although narrative in structure, the review incorporates systematic elements to enhance transparency and reproducibility.

A structured literature search was performed using the electronic databases PubMed, Web of Science, Scopus, ScienceDirect, SpringerLink, Google Scholar, and MDPI. Publications available up to November 2025 were considered. The search strategy included combinations of the following keywords: equine reproductive microbiota, uterine microbiota, vaginal microbiota, seminal microbiota, dysbiosis, endometritis, fertility, and 16S rRNA sequencing.

The initial search yielded approximately 312 records, of which 175 were screened based on title and abstract, and 127 were included following full-text evaluation. These steps ensured broad coverage of relevant literature while maintaining methodological rigor.

Studies were included if they met at least one of the following criteria: characterization of microbial communities within the equine reproductive tract (vaginal, uterine, penile, preputial, urethral, or seminal microbiota); application of culture-based, molecular, or sequencing-based methodologies; or investigation of associations between reproductive microbiota and reproductive physiology, fertility, or disease in horses. Preference was given to studies with clearly described sampling methodologies, appropriate contamination control strategies, and transparent analytical workflows, particularly in sequencing-based research.

Non-equine studies were included only when providing mechanistic or comparative context relevant to equine reproductive biology and were explicitly identified as such in the interpretation of findings.

Publications were excluded if they did not directly address reproductive microbiota, lacked sufficient methodological detail, or consisted exclusively of conference abstracts, non-peer-reviewed sources, or duplicate reports. No strict language restrictions were applied; however, priority was given to peer-reviewed publications in English. Titles and abstracts were screened for relevance, followed by full-text evaluation of eligible studies. Reference lists of included articles were manually screened to identify additional relevant publications.

Although formal risk-of-bias scoring was not performed, studies were critically appraised based on methodological rigor, including sampling design, contamination control, sequencing methodology, and analytical transparency. This qualitative appraisal ensured that methodological limitations were explicitly considered when interpreting findings.

Due to substantial methodological heterogeneity among studies, differences in sampling techniques, DNA extraction protocols, sequencing platforms, bioinformatic pipelines, and reporting standards—a quantitative meta-analysis was not feasible. Findings were therefore synthesized using a qualitative, thematic approach, with evidence organized according to anatomical location, methodological approach, reproductive phase, and clinical relevance.

This study did not involve animal experimentation or sample collection; therefore, ethical approval was not required.

## 3. Mare Reproductive System Anatomy, Estrous Cycle and Microbiota Localization

Accurate interpretation of the equine reproductive microbiota requires integrating anatomical, physiological, clinical, and methodological factors that regulate microbial distribution within the mare’s reproductive tract [12,15,38,39,40,41]. Structural barriers, including the vulvar seal, vestibulovaginal fold, and cervix, play a central role in limiting the ascension of microorganisms from the caudal reproductive tract into the uterus [38,40]. Barrier competence is influenced by age, parity, and perineal conformation, and compromised function is a recognized risk factor for microbial contamination, impaired uterine clearance, and endometritis [12,13,14,27,38,39,40,41,42].

The vagina harbors a relatively diverse microbial community, whereas the uterus is a low-biomass environment in which microbial detection is strongly influenced by sampling methodology, biomass yield, and contamination risk [15,16,17,20,21,22,23,24,25,26,43,44,45].

Sequencing-based studies have reported bacterial DNA within the uterus of clinically normal mares [16,17,45]; however, this finding should not be interpreted as definitive evidence of a stable resident microbiota, because uterine low-biomass samples are particularly vulnerable to contamination and sequencing-based methods do not distinguish viable from nonviable microorganisms [20,21,22,23,24,25,26]. Accordingly, interpretation of uterine microbial findings must remain conservative and method-aware. Different sampling approaches, including lavage-, swab-, cytobrush-, and biopsy-based methods, may yield non-equivalent microbial profiles and should not be considered analytically interchangeable [15,16,17,20,45]. These methodological differences are especially important when comparing studies or attempting to infer biological patterns.

Clinical reproductive evaluation provides essential context for interpreting microbiota findings. Assessment of perineal conformation, vaginal integrity, and cervical competence helps identify mares at increased risk of microbial ascension and impaired uterine clearance [38,39,40,41]. Transrectal ultrasonography enables detection of intrauterine fluid accumulation, while endometrial cytology and culture remain standard approaches for identifying inflammation and viable pathogens [12,27,46]. In contrast, sequencing-based approaches characterize total microbial DNA and therefore require cautious interpretation in low-biomass environments, where contaminant DNA and extracellular or nonviable microbial material may substantially distort observed microbial profiles [20,21,22,23,24,25,26]. Thus, clinical findings and sequencing results must be interpreted together rather than in isolation.

Hormonal fluctuations across the estrous cycle further modulate the reproductive tract environment and may influence microbial dynamics. The major endocrine phases of the estrous cycle and their relevance to microbial dynamics and uterine clearance are summarized in Table 1. During estrus, estrogen is associated with cervical relaxation, increased mucus secretion, and enhanced uterine clearance, conditions that may facilitate transient microbial exposure while favoring rapid elimination in healthy mares [12,27,38,40,47]. In contrast, progesterone-dominated diestrus is characterized by cervical closure, reduced myometrial activity, and altered endometrial responsiveness, conditions that may promote persistence of introduced microorganisms in susceptible mares [12,27,28,47,48,49]. However, direct causal relationships between endocrine regulation and microbiota composition in mares remain insufficiently defined, and many proposed mechanisms are inferred from physiological or comparative data rather than demonstrated directly in equine longitudinal microbiota studies [15,31,43,44]. Consequently, endocrine–microbiota interactions should be considered plausible but not yet empirically established in horses.

Expected microbiota patterns are inferred primarily from sequencing-based studies and physiological evidence and should be interpreted cautiously given the low microbial biomass of uterine samples, the absence of viability data, and the high susceptibility to contamination [20,21,22,23,24,25,26].

### 3.1. Vaginal Microbiota During Estrous Cycle in Mares

The vaginal microbiota of mares is a major microbial reservoir within the reproductive tract and a potential source of microorganisms that may ascend into the uterus [14,15,43,44,50]. Culture-based and sequencing-based approaches provide complementary but fundamentally different perspectives on this ecosystem and should be interpreted accordingly [15,20].

Sequencing-based studies consistently report that the equine vaginal microbiota is dominated at the phylum level by *Firmicutes*, *Bacteroidetes*, *Proteobacteria*, and *Actinobacteria* [43,44,50]. At finer taxonomic resolution, commonly reported genera include *Porphyromonas*, *Campylobacter*, *Corynebacterium*, *Streptococcus*, and *Fusobacterium* [43,44,50]. These findings indicate a relatively complex microbial community structure compared with culture-based observations, although sequencing-based profiles reflect total bacterial DNA and do not necessarily represent viable or metabolically active populations [20]. Thus, sequencing provides broad taxonomic resolution but limited insight into microbial viability or functional relevance.

In contrast, culture-based studies predominantly detect viable, fast-growing microorganisms, including *Escherichia coli*, *Streptococcus equi* subsp. *zooepidemicus*, and *Staphylococcus* spp. [14,50]. These taxa are frequently isolated from both clinically normal and affected mares and are particularly relevant in the context of endometritis, where they are commonly identified as opportunistic or pathogenic organisms [12,13,14]. This contrast underscores the methodological divergence between approaches and highlights the clinical relevance of culture-detected taxa.

A key distinction from the human vaginal microbiota is the relatively low abundance of *Lactobacillus* spp. in mares. Sequencing-based studies detect *Lactobacillus*-associated sequences in a minority of samples, while culture-based studies report variable isolation rates and demonstrate antimicrobial activity, adhesion capacity, and biofilm formation in vitro [51,52]. However, the functional role of these organisms in equine reproductive health remains unclear, and their relative scarcity does not necessarily indicate dysbiosis in the equine vaginal environment. Rather, it reflects species-specific physiological differences between equine and human vaginal ecosystems.

The extent to which the vaginal microbiota varies across the estrous cycle remains incompletely resolved. Some sequencing-based studies report relative stability between estrus and diestrus [43], while others suggest modest variation associated with hormonal status or environmental influences [44]. These inconsistencies likely reflect differences in study design, sampling strategy, sequencing approach, and bioinformatic analysis rather than definitive biological patterns. Overall, available evidence supports relative stability at higher taxonomic levels, with potential but insufficiently characterized variation at finer taxonomic resolution. Additionally, differences between studies may also reflect variation in sampling depth, anatomical site within the vagina, and environmental exposure, further complicating direct comparisons across datasets [15,20].

### 3.2. Uterine Microbiota During Estrous Cycle in Mares

The long-standing assumption that the healthy equine uterus is sterile has been increasingly challenged by sequencing-based studies reporting bacterial DNA in endometrial samples from clinically normal mares [16,17,29,45]. These findings suggest that the uterine environment may not be sterile in a strict microbiological sense; however, the biological significance of this signal remains uncertain, particularly given the low microbial biomass characteristic of uterine samples.

Sequencing-based investigations consistently detect bacterial DNA from the phyla *Proteobacteria*, *Firmicutes*, *Bacteroidetes*, and *Actinobacteria* [16,17,29,45,53]. At finer taxonomic resolution, genera such as *Streptococcus*, *Corynebacterium*, *Porphyromonas*, *Fusobacterium*, and *Escherichia*/*Shigella* are repeatedly identified [16,17,29,45]. While the recurrent detection of these taxa across studies suggests non-random microbial DNA profiles, it does not establish the presence of stable, resident microbiota. Notably, several taxa frequently reported in uterine sequencing datasets, including *Pseudomonas* and *Sphingomonas*, are also recognized as common contaminants in low-biomass microbiome studies, complicating interpretation of sequencing-derived data [21,22,23,24,25,26].

Recent paired-cycle sequencing data further support the presence of a structured microbial signal within the healthy equine uterus, showing that the endometrial microbiota of clinically normal mares was dominated by *Firmicutes*, *Proteobacteria*, *Bacteroidota*, and *Actinobacteriota*, which together accounted for more than 95% of total relative abundance [54]. At the genus level, *Staphylococcus*, *Acinetobacter*, *Sphingomonas*, *Corynebacterium*, *Streptococcus*, *Clostridium*, and *Pseudomonas* were among the most abundant taxa, reinforcing that microbial detection is reproducible across studies while still requiring cautious interpretation in low-biomass environments.

In contrast, culture-based studies provide a more conservative view of the uterine microbial environment. Classical investigations have shown that viable bacteria are infrequently recovered from the uterus of clinically normal mares, with many isolates likely originating from the caudal reproductive tract rather than representing true intrauterine colonization [13,14]. When detected, culture-based findings are typically dominated by fast-growing opportunistic organisms such as Escherichia coli and *Streptococcus equi* subsp. *zooepidemicus*, which are strongly associated with endometritis and impaired fertility [12,27]. This discrepancy reflects fundamental methodological differences: culture detects viable microorganisms capable of proliferation, whereas sequencing identifies total bacterial DNA, including nonviable cells and extracellular DNA fragments [19,20]. Thus, sequencing reveals potential microbial exposure, while culture identifies organisms with demonstrated viability and clinical relevance.

Cycle-dependent variation in uterine microbial profiles has been reported, although findings remain inconsistent [16,17,29,45]. Estrogen-dominated estrus is associated with cervical relaxation and enhanced uterine clearance, conditions that may facilitate transient microbial entry while limiting persistence in healthy mares [12,27,38,40,47]. In contrast, progesterone-dominated diestrus is characterized by cervical closure and altered endometrial responsiveness, which may promote persistence of introduced microorganisms in susceptible mares [12,27,28,48,49]. However, direct evidence linking endocrine fluctuations to specific microbiota changes in mares remains incompletely characterized, and observed differences may partly reflect methodological variability, including sampling strategy and sequencing approach, rather than true biological effects [16,17,45]. Consequently, endocrine-associated microbial patterns should be interpreted as plausible but not yet empirically validated.

Consistent with these physiological mechanisms, recent longitudinal paired sampling demonstrated significantly higher uterine alpha diversity during estrus compared with diestrus, while beta diversity showed substantial overlap between phases, indicating that the overall microbial structure remained relatively stable despite phase-dependent fluctuations [54]. Mare identity explained a substantially greater proportion of beta diversity variation than cycle phase, suggesting that inter-individual variation may outweigh hormonal effects when interpreting uterine microbiota profiles and may partially explain inconsistencies among studies.

A central unresolved question is whether microorganisms detected in the uterus represent true colonizers or transient signals arising from ascending migration, breeding, or sampling procedures. Studies using guarded transcervical sampling techniques and contamination-aware approaches provide more robust evidence for reproducible sequencing-derived signatures [16,17]; however, even under controlled conditions, distinguishing biologically relevant microorganisms from background contamination remains challenging [21,22,23,24,25,26]. High inter-individual variability further complicates interpretation and limits identification of consistent microbial patterns.

Overall, current evidence supports the presence of detectable bacterial DNA within the equine uterus but does not conclusively establish the existence of a stable, functionally active uterine microbiota. Interpretation of these findings requires a conservative, contamination-aware framework, particularly given the low microbial biomass of uterine samples, the risk of exogenous DNA contamination, and the inability of sequencing-based methods to distinguish viable from nonviable microorganisms [20,21,22,23,24,25,26]. Accordingly, the concept of a “core uterine microbiota” in mares should be considered provisional and highly dependent on methodological context, rather than definitively established. Representative studies investigating vaginal and uterine microbiota in mares, together with their principal methodological considerations and limitations, are summarized in Table 2.

Interpretation of microbiota data should explicitly consider methodological limitations, including low microbial biomass, contamination risk, the inability of sequencing-based approaches to distinguish viable from nonviable microorganisms, and variability in sampling, DNA extraction, and analytical pipelines [20,21,22,23,24,25,26].

Available studies collectively indicate that the mare’s reproductive tract is a hormonally responsive microbial environment, with the uterus representing a low-biomass niche [15,16,17,45,47]. Sequencing-based studies suggest relative stability at higher taxonomic levels across reproductive phases, while variation at finer taxonomic resolution has been reported between estrus, diestrus, and anestrus [16,17,45]. However, these patterns should be interpreted with caution, as they may reflect methodological variability rather than consistent biological trends. Thus, apparent cycle-associated differences may result from analytical rather than physiological factors.

In contrast, culture-based investigations predominantly identify viable opportunistic microorganisms, including taxa classically associated with endometritis, such as *Escherichia coli* and *Streptococcus equi* subsp. *zooepidemicus* [12,13,14,27]. Culture-based findings are influenced by endocrine status, sampling site, and anatomical location, but are limited to organisms capable of growth under laboratory conditions. Consequently, culture provides clinically relevant information on viable pathogens but captures only a subset of the microbial signal detected by sequencing.

Integration of culture-based and sequencing-based evidence indicates that the equine uterus contains detectable bacterial DNA and, in some cases, viable microorganisms; however, their ecological stability, persistence, and functional significance remain incompletely defined [16,17,29,45,53]. Importantly, sequencing-based detection must be interpreted cautiously in the context of low microbial biomass, where reagent-derived contamination and extracellular DNA may substantially influence observed microbial profiles [20,21,22,23,24,25,26]. Accordingly, standardized sampling strategies, rigorous contamination control, and transparent analytical reporting are essential to improve comparability across studies and to support accurate interpretation of uterine microbiota data in mares.

### 3.3. Methodological Considerations for Low-Biomass Uterine Samples

The equine uterus is a low-biomass microbial environment, making sequencing-based analyses particularly vulnerable to background contamination from sampling instruments, laboratory reagents, and environmental sources [21,22,23,25,26]. In these settings, contaminant DNA can comprise a substantial portion of the detected signal, complicating the distinction between true biological signal and methodological artifact.

Several taxa frequently reported in equine uterine microbiome studies—including *Ralstonia*, *Sphingomonas*, and *Pseudomonas*—are well-recognized reagent-associated contaminants in low-biomass sequencing datasets [21,22,23,25]. Their recurrent detection across studies does not necessarily indicate true uterine residency and must be interpreted cautiously, especially in the absence of appropriate negative controls.

Robust contamination control is essential to improve the reliability of microbiome data in this context. Recommended approaches include the use of negative sampling controls, extraction blanks, and sequencing blanks processed alongside biological samples, combined with computational methods such as prevalence-based filtering and contaminant identification algorithms [21,22,23,24,25]. Studies incorporating these measures provide stronger evidence for a reproducible uterine microbial signal, whereas studies lacking such controls are at increased risk of overestimating microbial diversity or misclassifying contaminants as biologically relevant taxa.

Another limitation of sequencing-based approaches is their inability to distinguish between viable and nonviable microorganisms [19,20]. This limitation is also evident in equine uterine studies comparing culture-based and sequencing-based findings [53]. Detection of bacterial DNA may therefore reflect lysed cells, residual extracellular DNA, or transient exposure following breeding, handling, or sampling, rather than active colonization [19,20]. This issue is particularly relevant in the equine uterus, where efficient post-breeding inflammatory responses and clearance mechanisms may eliminate viable bacteria while leaving detectable DNA signatures [27].

In addition to analytical constraints, sampling methodology substantially influences reported microbiota profiles [20]. Techniques such as low-volume lavage, cytobrush sampling, and endometrial biopsy differ in sampling depth, biomass recovery, and susceptibility to contamination, and may yield non-comparable microbial signatures [15,16]. Such methodological heterogeneity across studies further limits reproducibility and complicates cross-study comparisons.

Taken together, these methodological constraints necessitate a conservative, contamination-aware framework for interpreting uterine microbiota data in mares. Integrating sequencing-based approaches with culture-based diagnostics, viability-focused assays, and standardized sampling protocols will be essential to more accurately define the biological relevance of detected microorganisms and to distinguish true microbial colonization from transient or artifactual signals.

## 4. Dysbiosis and Reproductive Disorders in Mares

Dysbiosis refers to a disruption of the normal microbial equilibrium that alters host–microbe interactions and may compromise mucosal homeostasis [30]. In mares, dysbiosis has been associated with reduced reproductive efficiency; however, the strength and interpretation of this association depend strongly on the methodological approach. Culture-based studies have consistently linked uterine inflammation with the presence of opportunistic pathogens, while sequencing-based studies suggest that dysbiosis may involve broader alterations in microbial community structure rather than simply the presence or absence of specific organisms.

In equine reproduction, dysbiosis is best conceptualized as a spectrum of alterations that may include shifts in community composition, expansion of opportunistic or inflammation-associated taxa, reduced ecological stability, and disruption associated with external factors such as antimicrobial exposure. The principal forms of dysbiosis, representative microorganisms, proposed mechanisms, and reproductive consequences are summarized in Table 3.

Culture-based evidence provides the most consistent link between dysbiosis and reproductive pathology in mares [12,13,14,42,54]. Organisms such as *Streptococcus equi* subsp. *zooepidemicus*, *Escherichia coli*, and *Pseudomonas aeruginosa* are frequently isolated from mares with endometritis and are associated with impaired uterine clearance and persistent breeding-induced endometritis [12,13,14,27,42]. These microorganisms are commonly present in the caudal reproductive tract, particularly the vestibule and clitoral fossa, supporting the concept that dysbiosis may involve ascension and overgrowth of opportunistic taxa rather than the introduction of exogenous pathogens [13,14]. This pattern aligns with the broader view that equine reproductive dysbiosis reflects ecological imbalance rather than invasion by novel pathogens.

Importantly, genera traditionally associated with equine endometritis, including Staphylococcus, Streptococcus, and Pseudomonas, have also been identified among dominant taxa in clinically healthy mares [54]. This finding emphasizes that interpreting dysbiosis should not rely solely on the presence of individual microorganisms, but rather on changes in microbial structure, abundance patterns, host inflammatory status, and uterine clearance capacity.

It is important to distinguish between post-breeding endometritis (PBE) and persistent breeding-induced endometritis (PBIE), as these conditions represent fundamentally different biological processes. Post-breeding endometritis is a normal, transient physiological inflammatory response that occurs after natural mating or artificial insemination and serves to eliminate excess spermatozoa, seminal plasma components, and introduced microorganisms from the uterine lumen [27,28]. In healthy mares, this inflammatory response is rapidly resolved through effective uterine contractions, neutrophil recruitment, lymphatic drainage, and cervical relaxation, typically within 24 to 48 h after breeding [12,27,55].

In contrast, persistent breeding-induced endometritis is a pathological failure of this clearance process, characterized by prolonged inflammation, delayed resolution, intrauterine fluid accumulation, and increased susceptibility to opportunistic bacterial persistence [12,27,28]. PBIE is particularly common in older, multiparous, or anatomically compromised mares with impaired uterine clearance mechanisms.

In this context, microorganisms such as *Streptococcus equi* subsp. *zooepidemicus*, *Escherichia coli*, and *Pseudomonas aeruginosa* may persist and contribute to chronic inflammation and subfertility. Dysbiosis should therefore be interpreted primarily in relation to impaired uterine defense and failure of physiological clearance, rather than as a simple consequence of bacterial presence alone.

In contrast, the role of “beneficial” microorganisms in mares remains less clearly defined than in other species. Unlike the human vaginal microbiota, which is typically dominated by *Lactobacillus* spp., equine studies consistently demonstrate a low relative abundance of lactic acid–producing bacteria [43,51]. Although Lactobacillus and *Enterococcus* spp. may exhibit antimicrobial activity in vitro [51], their functional contribution to reproductive tract homeostasis in mares remains uncertain. Consequently, dysbiosis in mares is unlikely to be driven solely by the loss of a single protective taxon and is better interpreted as a shift in overall microbial balance [56]. This species-specific ecological context is essential for avoiding inappropriate extrapolation from human or bovine models.

Sequencing-based studies further support the concept that dysbiosis may occur at the level of community structure rather than major taxonomic shifts. Recent equine studies indicate that mares with clinical endometritis may exhibit uterine microbial profiles characterized by increased relative abundance of taxa such as *Streptococcus* or *Escherichia/Shigella*, with alterations in diversity observed following intrauterine antibiotic treatment [29,53]. Core phyla, including *Firmicutes*, *Bacteroidetes*, *Proteobacteria*, and *Actinobacteria*, are consistently detected across reproductive states [16,17,45]. However, interpretation remains constrained by low microbial biomass, contamination risk, and the inability of sequencing-based approaches to determine microbial viability. Thus, sequencing-based signatures of dysbiosis should be viewed as indicators of altered microbial DNA patterns rather than definitive evidence of active microbial proliferation.

External factors, particularly antimicrobial exposure, represent an additional driver of dysbiosis. Repeated or empirical intrauterine antibiotic use may disrupt commensal microbial communities and promote selection of resistant or opportunistic organisms [57]. Evidence also indicates that antibiotic exposure can alter the vaginal microbiota and contribute to antimicrobial resistance development in mares [50,58]. While targeted antimicrobial therapy remains essential for treating confirmed infections, inappropriate or excessive use may exacerbate microbial imbalance. This highlights the need for judicious antimicrobial stewardship in equine reproductive practice.

Overall, dysbiosis in the equine reproductive tract reflects a shift from a relatively stable, host-regulated microbial environment toward increased dominance of opportunistic or inflammation-associated taxa. These changes have been associated with impaired uterine homeostasis, inflammation, and reduced fertility; however, causality remains difficult to establish. Future research should prioritize longitudinal, contamination-aware, and functionally integrated study designs to clarify whether dysbiosis represents a primary driver of reproductive pathology or a secondary consequence of impaired uterine function.

**Table 3:** Key forms of dysbiosis in mares, representative microorganisms, mechanisms, and reproductive implications.

Form of Dysbiosis	Representative Microorganisms	Main Mechanisms	Reproductive Consequences
Loss of beneficial commensals	*Lactobacillus*, *Enterococcus*	Reduced production of antimicrobial metabolites; decreased competitive exclusion; altered mucosal environment	Potential increased susceptibility to opportunistic colonization (limited direct equine evidence) [51,52]
Overgrowth of opportunistic or pathogenic taxa	*E. coli*, *S. equi* subsp. *zooepidemicus*, *Pseudomonas*, *Fusobacterium* spp.	Impaired uterine clearance, inflammatory activation, microbial overgrowth	Endometritis, PBIE, reduced fertility [12,13,14,27,42]
Reduced microbial diversity and instability	No consistently enriched taxa	Loss of ecological resilience; increased community instability; altered host–microbe interactions	Potential association with chronic inflammation and subfertility (conceptual, extrapolated from general microbiome theory) [30]
Antibiotic-associated dysbiosis	Resistant or opportunistic taxa	Disruption of commensal communities; selective pressure favoring resistant organisms	Recurrent infection; altered microbial profiles; delayed restoration of microbial balance [55,57]
Emerging biomarkers	Microbial community signatures; host immune markers (e.g., cytokines)	Early detection of microbial imbalance; integration of host–microbiome interactions	Potential diagnostic and management applications (currently limited equine validation) [15,45]

Culture-based studies identify viable microorganisms capable of growing under laboratory conditions and have consistently linked opportunistic taxa with reproductive pathology in mares, whereas sequencing-based approaches detect total microbial DNA, including that from nonviable cells and extracellular material. In low-biomass environments such as the equine uterus, sequencing-derived profiles are particularly susceptible to contamination and analytical bias. Consequently, definitions and interpretations of dysbiosis in mares must be evaluated within the methodological context of the approach used.

## 5. Stallion Reproductive Tract Microbiota and Reproductive Implications

Stallion reproductive health directly affects herd fertility and plays a significant role in the transmission of venereal pathogens [5,6,27,36]. Although historically less studied than mares, growing evidence indicates that the stallion reproductive tract harbors a detectable microbial signal that may be associated with semen quality and reproductive outcomes [32,33,34,37].

### 5.1. Anatomical and Semen-Related Context

The stallion reproductive tract—including the penis, prepuce, urethra, epididymis, and accessory sex glands—provides the anatomical and biochemical environment where sperm and microorganisms coexist [5,59,60]. While these structures influence seminal plasma composition, current evidence suggests that anatomical features alone have a limited direct impact on seminal microbial profiles compared to the more pronounced anatomical–microbial interactions observed in mares.

Instead, microorganisms detected in semen are thought to primarily originate from the distal reproductive tract, particularly the prepuce and urethra, as well as from external sources associated with semen collection [34,36,61,62,63]. This underscores the importance of sampling conditions, as collection using an artificial vagina introduces multiple potential sources of contamination, including equipment, environment, and handling procedures. Therefore, methodological rigor is essential for accurate interpretation of seminal microbiota data.

### 5.2. Semen Quality and Microbiota Interpretation

Standard semen evaluation parameters—including sperm concentration, motility, morphology, and viability—provide essential context for interpreting seminal microbiota findings, as stallion fertility is influenced by multiple interacting factors, including genetic background, reproductive tract health, and management conditions [5,7]. Microorganisms detected in semen may originate from the urethra, preputial cavity, or external genitalia, and distinguishing commensal organisms from clinically relevant pathogens requires integrating microbiological findings with semen quality data [32,36,61].

Culture-based methods identify viable microorganisms and enable antimicrobial susceptibility testing but are inherently biased toward fast-growing aerobic or facultative taxa [61,64]. In contrast, sequencing-based approaches, particularly 16S rRNA gene sequencing, provide broader characterization of microbial DNA, including anaerobic, fastidious, and low-abundance taxa not detectable by culture [32,33,34,37]. These methodological differences significantly influence reported microbial composition and complicate comparisons across studies, highlighting the need for standardized sampling and analytical protocols. Without such standardization, cross-study variability may reflect methodological artifacts rather than true biological differences.

### 5.3. Composition of the Stallion Seminal Microbiota

Both culture-based and sequencing-based studies show that stallion semen contains a diverse assemblage of microorganisms, including commensal taxa, opportunistic organisms, and recognized reproductive pathogens [32,33,34,36,37,64]. A comparative summary of seminal microbiota composition identified by different methodological approaches is presented in Table 4.

Culture-based investigations commonly isolate *Staphylococcus* spp., *Micrococcus* spp., *Escherichia coli*, *Streptococcus equi* subsp. *zooepidemicus*, *Pseudomonas* spp., and *Klebsiella* spp. [61,62,63,64]. Reported isolates frequently include coagulase-negative *staphylococci*, *coryneform bacteria*, *Streptococcus* spp., *Escherichia coli*, *Pseudomonas* spp., and *Klebsiella* spp., reflecting both commensal colonization of the distal reproductive tract and potential opportunistic pathogens relevant to reproductive disease [61,62,63,65]. These organisms are frequently considered commensals of the external genitalia but may act as opportunistic pathogens when introduced into the uterus, particularly in susceptible mares [12,13,14,27,28,42,55,66].

Sequencing-based studies have broadened this perspective by identifying dominant phyla such as *Firmicutes*, *Bacteroidetes*, *Actinobacteria*, and *Proteobacteria* [32,33,34,37]. At finer taxonomic resolution, families including *Porphyromonadaceae*, *Peptoniphilaceae*, *Corynebacteriaceae*, and *Prevotellaceae* are consistently detected across studies, suggesting non-random microbial DNA profiles. However, the concept of a stable “core seminal microbiota” remains provisional, as most studies are cross-sectional, show substantial inter-individual variability, and are susceptible to contamination introduced during semen collection and processing.

This variability is influenced by multiple factors, including stallion-specific characteristics, management practices, environmental exposure, and sampling methodology [34,63,67,68]. Contamination from the environment or collection process is a significant confounding factor, particularly in sequencing-based analyses, and must be considered when interpreting microbial profiles.

Emerging evidence suggests potential associations between specific microbial taxa and semen quality. For example, members of the *Peptoniphilaceae* family have been associated with higher sperm motility, while certain *Clostridiales* taxa have been reported to negatively correlate with progressive motility [32,33,34,37]. However, these associations remain inconsistent through studies, and causal relationships have not been established. Differences in study design, sample size, and analytical methodology likely contribute to these inconsistencies.

### 5.4. Clinical and Reproductive Implications

The seminal microbial community may influence reproductive outcomes by directly affecting sperm quality or by serving as a source of microorganisms transmitted to mares during natural breeding or artificial insemination [5,6,36,60,61,62,69,70]. Bacterial contamination has been associated with reduced sperm motility and viability, especially during semen storage, where microbial proliferation may occur despite the use of antibiotics in extenders.

Alterations in seminal microbial composition have also been observed in stallions carrying venereal pathogens such as *Taylorella equigenitalis*, suggesting potential interactions between pathogen carriage and the broader microbial community [36]. However, the extent to which these interactions influence fertility remains unclear.

Although the stallion’s role as a source of microbial transmission to mares is widely recognized, direct longitudinal evidence linking specific stallion-derived strains to persistent colonization or dysbiosis in mares remains limited. Most available data come from cross-sectional or culture-based studies, which restrict strain-level resolution and limit mechanistic interpretation.

Current evidence indicates that stallion semen contains diverse and dynamic sequencing-derived signatures shaped by host, environmental, and methodological factors. While its clinical relevance is increasingly recognized, substantial knowledge gaps remain regarding its functional role, ecological stability, and contribution to fertility outcomes. Future studies integrating culture-based diagnostics, high-resolution sequencing, and functional approaches—particularly in longitudinal, paired stallion–mare designs—will be essential to clarify the role of the seminal microbiota in equine reproduction.

Interpretation of seminal microbiota data should explicitly consider methodological limitations, including contamination risk, the inability to distinguish viable from nonviable microorganisms, and variability in sampling and analytical protocols. These constraints are especially relevant when comparing findings from culture-based and sequencing-based approaches.

Stallion semen has been shown to contain diverse microbial DNA profiles influenced by fertility status, pathogen carriage, and environmental and management factors [6,34,36,37,62,64,67,68]. Sequencing-based (NGS) studies consistently report broader and more complex microbial DNA profiles, including anaerobic and low-abundance taxa, while culture-based methods primarily identify viable, fast-growing aerobic or facultative microorganisms [32,33,34,61,62,64]. These methodological differences significantly affect reported microbial composition and limit direct comparability across studies. Therefore, cross-study variation may reflect analytical artifacts as much as true biological differences.

Overall, current evidence supports the presence of reproducible but highly variable microbial DNA signatures rather than purely incidental contamination; however, their biological relevance remains incompletely defined. Although associations between specific microbial taxa and sperm function or reproductive outcomes have been reported, these relationships are inconsistent across studies and remain largely correlative rather than causal [6,32,33,34,37,67]. Thus, microbiota–semen quality associations should be viewed as preliminary indicators rather than established mechanistic links. Integration of microbiological findings with semen quality parameters and standardized, contamination-aware study designs is essential to clarify the functional significance of seminal microbiota in equine reproduction.

## 6. Seminal–Uterine Microbial Transmission Between Stallions and Mares

Microbial exchange between stallions and mares during natural mating or artificial insemination is a biologically plausible mechanism influencing reproductive outcomes; however, direct evidence for sustained microbial transmission and colonization remains insufficiently defined [5,36,60,61,62,63].

Current understanding comes from a combination of culture-based studies, sequencing-based profiling, and indirect observational evidence, each with significant methodological constraints that complicate interpretation, especially in low-biomass microbiome research [15,20,21,22,23,24].

Culture-based studies provide direct evidence that viable microorganisms are present in stallion ejaculates and can be introduced into the mare reproductive tract during breeding [61,62,63,64]. Bacteria commonly isolated from semen collected with an artificial vagina include coagulase-negative *staphylococci*, *coryneform bacteria*, *streptococci*, and, in some cases, opportunistic or venereal-associated organisms such as *Klebsiella pneumoniae* and *Pseudomonas aeruginosa* [61,62,63,64]. These findings support the concept of active microbial transfer; however, culture-based methods are inherently biased toward fast-growing aerobic and facultative organisms and therefore offer only a partial representation of the seminal microbial community [15,20,61,64].

Sequencing-based studies further show that stallion semen harbors structured microbial DNA profiles dominated by *Firmicutes*, *Bacteroidetes*, *Proteobacteria*, and *Actinobacteria* [32,33,34,35,37]. At finer taxonomic resolution, genera such as *Porphyromonas*, *Peptoniphilus*, and *Corynebacterium* have been repeatedly detected across independent studies [32,33,34,35,37]. Although repeated identification of these taxa suggests a non-random microbial signal, interpretation remains limited because sequencing-based approaches detect total microbial DNA regardless of viability and may also capture environmental or reagent-derived contamination [20,21,22,23,24]. Consequently, the extent to which sequencing-derived seminal profiles represent biologically active and transmissible microbial communities remains uncertain.

Within the mare, the vaginal microbiota is the primary interface for semen-associated microorganisms and may serve as a microbial reservoir and a selective ecological interface [15,43,44,50]. Sequencing-based studies indicate relative stability of the vaginal microbiota at higher taxonomic levels across the estrous cycle [43,44], while culture-based studies show variable recovery of opportunistic organisms, including *Escherichia coli* and *Streptococcus equi* subsp. *zooepidemicus*, depending on reproductive phase and local conditions [14,44,50,58].

Hormonal regulation may further influence susceptibility to microbial persistence, as progesterone-dominated conditions are associated with reduced uterine clearance, decreased myometrial contractility, and altered endometrial immune responsiveness in mares [12,27,28,47,48,49]. However, direct equine evidence linking endocrine state to microbial transmission dynamics remains limited, and many mechanistic interpretations are extrapolated from broader reproductive microbiome literature rather than demonstrated experimentally in horses [31,71,72].

A central unresolved question is whether semen-associated microorganisms establish persistent colonization within the mare reproductive tract or instead represent transient exposure followed by effective uterine clearance [12,27,28,55]. Evidence supporting stallion-to-mare transmission is largely based on the detection of similar bacterial taxa in semen and post-breeding samples from the mare reproductive tract [14,36,61,62,63]. However, these observations come mainly from culture-based identification or non-paired study designs and therefore lack strain-level resolution and temporal context [36,61,62,63]. As a result, it is not possible to distinguish true inter-host transmission from coincidental detection of common commensal, opportunistic, or environmentally acquired taxa [20,21,22,23,24,36].

Although sequencing-based methods provide broader taxonomic coverage, they have rarely been used in longitudinal paired stallion–mare study designs, limiting inference regarding directionality, persistence, and functional relevance of detected microorganisms [32,33,34,35,37]. Interpretation is further complicated by the low microbial biomass of the equine uterus, where detected DNA may represent transient contamination, extracellular DNA, or nonviable cells rather than stable colonization [15,16,17,20,21,22,23,24,45].

Reciprocal transmission from mares to stallions during natural mating is biologically plausible but remains poorly characterized due to the absence of adequately designed longitudinal studies [36,62,63].

The outcome of microbial exposure during breeding is strongly influenced by host factors, particularly the mare’s uterine defense mechanisms [12,27,28,42,55]. It is important to distinguish physiological post-breeding endometritis (PBE) from persistent breeding-induced endometritis (PBIE). PBE is a normal, transient inflammatory response after mating or insemination that facilitates the clearance of spermatozoa, seminal plasma, and introduced microorganisms. In contrast, PBIE is characterized by pathological persistence of inflammation due to impaired uterine clearance, delayed resolution, and increased susceptibility to opportunistic bacterial persistence [12,27,28]. This distinction is critical when determining whether semen-associated microorganisms represent transient exposure or clinically relevant microbial persistence. In healthy mares, post-breeding inflammation, uterine contractility, and efficient clearance mechanisms typically eliminate introduced microorganisms within a short period after mating or insemination [12,27,28,42,55]. In contrast, mares susceptible to persistent breeding-induced endometritis show impaired innate immune responses, delayed resolution of inflammation, and reduced uterine clearance, which may allow persistence of opportunistic microorganisms [12,27,28,55]. Additional factors, such as age-related anatomical changes, reduced myometrial contractility, and compromised lymphatic drainage, may further predispose mares to microbial persistence and chronic uterine pathology [12,27,28,42].

Post-breeding inflammation is not exclusively microbe-driven and may also be triggered by spermatozoa and seminal plasma components [12,13,27,28]. Opportunistic pathogens such as *Pseudomonas aeruginosa*, *Klebsiella pneumoniae*, and *Streptococcus equi* subsp. *zooepidemicus* may persist when uterine defense mechanisms are impaired and are consistently associated with endometritis in mares [12,13,14,28,42,54,66]. Detection of these organisms within the endometrium suggests potential mechanisms of persistence; however, whether this reflects active colonization, biofilm formation, or passive retention remains unclear [12,66].

**Figure 1:**
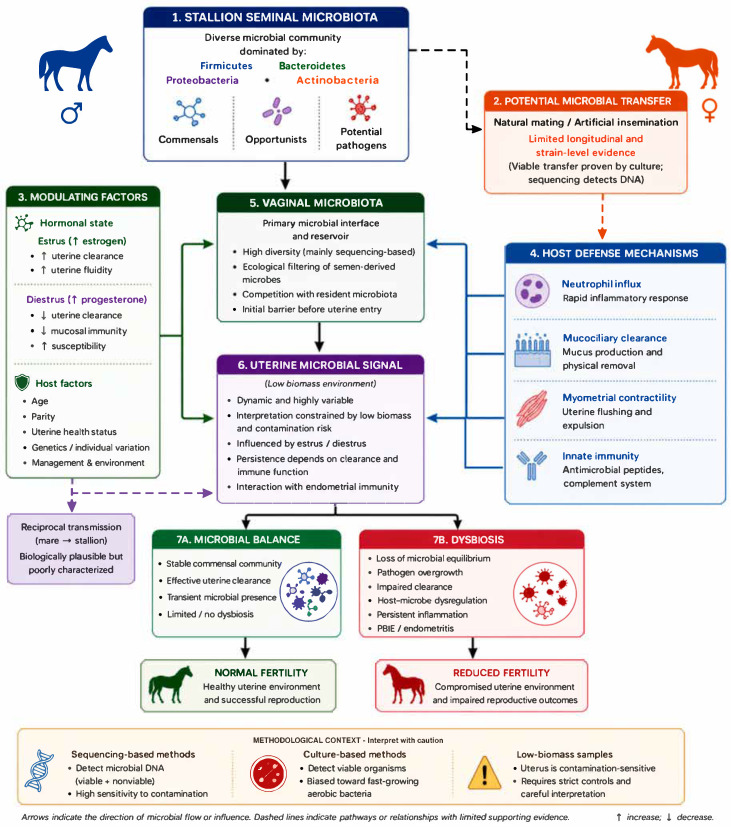
Conceptual framework of semen-associated microbial transfer, host–microbe interactions within the mare reproductive tract, and potential reproductive outcomes.

Overall, available evidence supports the concept that microbial exchange between stallions and mares occurs during breeding, but its ecological stability, persistence, and clinical significance remain incompletely defined [5,12,15,32,33,34,35,36,37,61,62,63]. Interpretation is limited by the predominance of cross-sectional studies, the absence of strain-resolved paired designs, and methodological challenges inherent to low-biomass microbiome research [15,20,21,22,23,24]. Future studies using longitudinal paired stallion–mare sampling, combined with high-resolution sequencing, viability-oriented methods, and functional analyses, will be needed to distinguish transient microbial transfer from biologically meaningful colonization and to clarify its role in equine reproductive health [15,20,32,33,34,35,37,73,74]. These processes and their methodological constraints are summarized in the conceptual framework (Figure 1).

Semen-associated microorganisms introduced during natural mating or artificial insemination may interact with the vaginal microbiota, which serves as the primary microbial interface and a selective ecological barrier before potential uterine entry. The mare reproductive tract is a hormonally regulated system in which microbial exposure is modulated by anatomical barriers, endocrine status, and uterine defense mechanisms.

Within the uterus, a low-biomass environment, microbial persistence depends on the balance between microbial load and host-mediated clearance processes, including post-breeding inflammation, myometrial contractility, and innate immune responses. In healthy mares, these mechanisms typically enable rapid elimination of introduced microorganisms, while impaired clearance may permit persistence of opportunistic taxa and contribute to conditions such as persistent breeding-induced endometritis.

Interpretation of uterine microbial profiles is further limited by methodological factors. Sequencing-based approaches detect total microbial DNA and are therefore affected by contamination, low microbial biomass, and the inability to distinguish viable from nonviable microorganisms. As a result, detected microbial signals may reflect transient exposure, residual extracellular DNA, or methodological artifacts rather than stable colonization.

## 7. Discussion

This review synthesizes current evidence on the equine reproductive microbiota and indicates that the reproductive tracts of mares and stallions are associated with detectable microbial communities or sequencing-derived microbial signals that may interact with host physiology. However, the strength of evidence linking specific microbiota patterns to reproductive function remains variable, and interpretation strongly depends on methodological context.

Across studies, the vaginal microbiota of mares consistently exhibits a relatively stable phylum-level structure dominated by *Firmicutes*, *Bacteroidetes*, *Proteobacteria*, and *Actinobacteria* [16,43,44,50]. In contrast, interpretation of uterine microbiota is considerably more complex due to the low microbial biomass of endometrial samples and the associated susceptibility to contamination [21,22,23,25,26]. At finer taxonomic resolution, substantial genus- and species-level variability is reported, reflecting the combined influence of reproductive phase, endocrine status, anatomical barriers, and methodological heterogeneity among studies [17,18,29,44,45]. Although associations between reduced microbial diversity, enrichment of opportunistic taxa, and reproductive disorders such as endometritis have been described, these associations are primarily observational and do not establish causality [12,27,28,56].

A major source of variability in literature arises from fundamental differences between culture-based and sequencing-based methodologies. Culture-based approaches identify viable, fast-growing microorganisms and remain essential for pathogen detection and antimicrobial susceptibility testing, but they inherently underestimate microbial diversity and fail to capture anaerobic or fastidious taxa [61,62,63]. In contrast, sequencing-based approaches provide broader community-level characterization but detect microbial DNA regardless of viability and are highly sensitive to contamination in low-biomass environments such as the equine uterus [20,21,22,23,24]. Consequently, apparent discrepancies between studies—such as stable phylum-level profiles identified by sequencing versus fluctuating opportunistic pathogens detected by culture—are more likely to reflect methodological differences rather than true biological inconsistency [16,43,44,50]. Interpretation of microbiota data therefore requires explicit consideration of detection method, viability, and contamination risk. Standardized sampling protocols, rigorous inclusion of negative controls, and harmonized analytical pipelines are essential to improve reproducibility and cross-study comparability.

Hormonal regulation is likely to influence microbial dynamics within the reproductive tract, although direct mechanistic evidence in mares remains incompletely characterized. Estrus is characterized by increased cervical patency, enhanced mucosal secretions, and active innate immune responses, including neutrophil recruitment and uterine contractility, which collectively facilitate microbial clearance [12,27,48]. In contrast, progesterone-dominated diestrus is associated with reduced uterine contractility, altered immune responsiveness, and decreased clearance capacity, potentially allowing persistence of introduced microorganisms [47,48,49]. While sequencing-based studies report phase-dependent variation in certain taxa, findings remain inconsistent and may be influenced by sampling strategy and analytical approach [17,45]. Mechanistic insights from other species suggest that endocrine modulation of mucosal immunity and epithelial barrier function may influence microbial composition; however, the applicability of these mechanisms to equine reproductive physiology remains to be established [31].

The role of dysbiosis in equine reproductive disorders remains an area of active investigation. Persistent detection of opportunistic organisms such as *Escherichia coli* and *Streptococcus equi* subsp. *zooepidemicus* is consistently associated with post-breeding endometritis, a major cause of subfertility in mares [12,13,14,42,54,66]. Dysbiosis—defined as disruption of commensal microbial balance, expansion of opportunistic taxa, and reduced ecological stability [30]—may contribute to impaired uterine defense and chronic inflammation. However, whether dysbiosis represents a primary etiological driver or a secondary consequence of an altered uterine environment remains unresolved.

Microbial dysbiosis may also contribute indirectly to reproductive dysfunction through inflammatory and oxidative pathways. In mares, persistent uterine inflammation is associated with increased production of reactive oxygen species, which can impair endometrial function and disrupt cellular homeostasis [12,28]. In stallions, oxidative stress is a well-established determinant of sperm quality, affecting membrane integrity, motility, and DNA stability [69,70]. Although microbiota–oxidative stress interactions have been demonstrated in other species, they remain insufficiently characterized in horses.

From a clinical perspective, microbiota profiling should currently be considered a complementary rather than a primary diagnostic tool in equine reproduction [15]. Culture-based diagnostics remain essential for identifying viable pathogens and guiding antimicrobial therapy, while sequencing-based approaches provide broader ecological context but require cautious interpretation. Detection of bacterial DNA alone does not justify antimicrobial intervention, particularly in the absence of cytological or clinical evidence of inflammation. Antimicrobial treatment may substantially alter uterine microbial composition, with equine studies demonstrating shifts in diversity and dominant taxa following intrauterine antibiotic administration [53]. However, whether these changes represent restoration of microbial balance or treatment-induced dysbiosis remains unclear. Integration of microbiological findings with clinical examination, ultrasonography, cytology, and reproductive history is critical for accurate diagnosis and management. Inappropriate or empirical antimicrobial use may disrupt commensal microbial communities and contribute to antimicrobial resistance [75].

The stallion seminal microbiota is an integral component of the reproductive microbial system and may influence fertility through both direct effects on sperm quality and indirect effects via microbial transmission to mares [32,33,34,37]. While antibiotics in semen extenders effectively reduce bacterial load, they may also alter microbial composition and contribute to antimicrobial resistance [75,76,77]. Alternative approaches, including improved hygienic practices, selective antimicrobial use, and physical bacterial reduction techniques such as filtration or colloidal centrifugation, may reduce microbial load while preserving microbial balance [55,60].

Overall, the equine reproductive microbiota should be conceptualized as a dynamic, host-associated system shaped by interactions among microbial communities, endocrine regulation, immune responses, and management practices. Importantly, equine reproduction involves a coupled microbial system encompassing both mare and stallion rather than independent biological units. Current evidence supports the occurrence of microbial exchange during breeding; however, its persistence, ecological stability, and clinical relevance remain incompletely defined. Future research should prioritize longitudinal, paired stallion–mare study designs incorporating high-resolution sequencing, viability-based approaches, and multi-omics integration to distinguish transient microbial exposure from biologically meaningful colonization and to clarify the role of reproductive microbiota in equine fertility [74].

## 8. Future Perspectives

Future research on the equine reproductive microbiota should move beyond descriptive characterization toward mechanistic, longitudinal, and clinically translatable investigations. Although current studies demonstrate the presence of detectable microbial communities or sequencing-derived microbial signals within the reproductive tracts of mares and stallions, the functional roles of specific taxa and their causal relationships with fertility remain largely unresolved.

A key priority is the implementation of longitudinal, paired stallion–mare study designs in which both partners are sampled before and after breeding. Such approaches would enable direct assessment of microbial exchange, persistence, and temporal dynamics, while improving inference regarding transmission directionality and ecological stability. Integration of high-throughput sequencing with host immune, endocrine, and transcriptomic profiling may further clarify host–microbe interactions; however, distinguishing biologically meaningful signals from transient microbial exposure and methodological artifacts will remain a critical challenge.

Standardization of sampling strategies, DNA extraction protocols, and bioinformatic pipelines is essential to improve comparability across studies. In particular, the low microbial biomass of the equine uterus necessitates rigorous contamination-aware methodologies, including routine use of negative controls, extraction blanks, and statistical decontamination approaches. Adoption of recently proposed reporting and quality-control frameworks for low-biomass microbiome research may further enhance reproducibility and transparency. In parallel, incorporation of viability-oriented approaches, such as culture integration or viability PCR, may help differentiate living microorganisms from nonviable cells or extracellular DNA, although the biological relevance of nonviable microbial components should not be overlooked.

While this review has focused primarily on bacterial communities, other components of the reproductive microbiome—including fungi, archaea, and viruses—remain largely unexplored in horses. Expanding research toward multi-kingdom and multi-omics approaches will be necessary to achieve a more comprehensive understanding of microbial ecosystem structure and function within the reproductive tract.

From a clinical perspective, microbiota-informed approaches may contribute to improved risk stratification and management of reproductive disorders in mares, including persistent breeding-induced endometritis, embryo loss, and subfertility. However, current evidence does not support the use of microbiota profiling as a standalone diagnostic tool. Microbial data should be interpreted in conjunction with cytology, ultrasonography, endocrine assessment, and reproductive history. Future controlled studies are required to evaluate the efficacy and safety of microbiota-modulating interventions, including probiotics, prebiotics, bacteriophages, and selective antimicrobial strategies [63]. Preliminary findings suggest that targeted microbial modulation may be feasible; however, robust equine-specific evidence remains limited.

In stallions, future research should evaluate how semen collection, processing, and preservation techniques influence the seminal microbiota and its interaction with sperm function. The development of antibiotic-sparing or antibiotic-free extenders, alongside physical bacterial reduction methods such as filtration or colloidal centrifugation, represents a promising direction for reducing reliance on broad-spectrum antimicrobials while preserving semen quality and microbial balance.

A major future challenge will be distinguishing normal physiological variation and mare-specific microbial signatures from microbiota alterations associated with reproductive disease, particularly in low-biomass uterine environments where methodological artifacts may strongly influence interpretation [54]. This distinction will be essential for developing clinically meaningful diagnostic thresholds and avoiding overinterpretation of sequencing-based findings.

Finally, integration of microbiome data with endocrine, genetic, and reproductive performance parameters may support the development of individualized, evidence-based reproductive management strategies [7]. However, translation of microbiome research into clinical practice will require robust validation in well-controlled studies, including demonstration of reproducibility, biological relevance, and measurable clinical benefit. Progress in this field will therefore depend not only on technological innovation but also on rigorous study design and critical, contamination-aware interpretation of microbiome data within the unique context of equine reproductive physiology.

## 9. Conclusions

Current evidence indicates that the equine reproductive tract cannot be considered consistently sterile when assessed using sequencing-based methodologies but is associated with detectable microbial communities or sequencing-derived microbial signals, the interpretation of which depends strongly on methodological context. Culture-based and sequencing-based approaches provide complementary but fundamentally different perspectives, capturing viable microorganisms and total microbial DNA, respectively.

In mares, vaginal microbiota shows relative stability at the phylum level, whereas interpretation of uterine microbial findings is constrained by low microbial biomass, contamination risk, and limited ability to distinguish viable from nonviable microorganisms. In stallions, semen contains a diverse microbial assemblage that may influence sperm quality and represents a potential source of microbial exposure during breeding.

Associations between microbial alterations and reproductive disorders, including persistent breeding-induced endometritis, are supported by observational evidence; however, causal relationships remain unproven. Microbiota data should therefore be interpreted within the broader framework of host–microbe interactions, uterine clearance mechanisms, endocrine regulation, and host genetic factors influencing reproductive performance.

Overall, the equine reproductive microbiota represents a dynamic and context-dependent system shaped by interactions between microbial communities, host physiology, and management practices. Progress in this field will require standardized, contamination-aware methodologies, longitudinal and paired study designs, and integration of microbiome data with clinical and functional outcomes to establish biological relevance and support evidence-based reproductive management.

## Figures and Tables

**Table 1:** Key endocrine states and their relevance to microbial dynamics in the mare’s reproductive tract.

Reproductive Phase	Dominant Hormones	Microbiota-Relevant Physiological Changes	Expected Microbiota Pattern	Clinical Relevance/References
Estrus	High estradiol (E2)	Cervical relaxation; increased mucus secretion; increased uterine contractility and clearance	Higher likelihood of transient microbial exposure from the caudal tract, but limited persistence in healthy mares	Exposure may increase, but effective clearance usually limits sustained microbial persistence [12,27,38,40,47]
Diestrus	Progesterone (P4) dominance	Cervical closure; reduced myometrial activity; altered endometrial responsiveness; more static intrauterine environment	Greater potential persistence of introduced microorganisms in susceptible mares	Increased susceptibility to PBIE and persistence of opportunistic bacteria when uterine defense mechanisms are impaired [12,27,28,47,48,49]
Transitional periods	Fluctuating E2; inconsistent luteal support	Variable cervical tone; inconsistent uterine clearance; endocrine instability	Increased variability in microbial detection; interpretation is strongly method-dependent	May predispose to impaired clearance and inconsistent reproductive tract findings, although direct microbiota data remain limited [46,47]
Across-cycle variation	Involves changing E2:P4 ratios and dynamics	Changes in mucus, uterine tone, and immune activity	Phase-dependent variation is plausible, but high inter-individual variability and methodological effects complicate interpretation	This may contribute to variation in susceptibility to inflammation and reproductive outcomes, but current evidence remains mainly observational [12,15,43,44,45]

**Table 2:** Summary of key equine studies investigating vaginal and uterine microbiota in mares.

Study	Site	Method	Cycle Phases	Key Findings and Methodological Considerations	Limitations
Barba et al., 2020 [43]	Vagina	Sequencing + culture	Estrus/Diestrus	Relative stability at the phylum level across the cycle; low *Lactobacillus* abundance; sequencing reveals greater diversity than culture-based approaches	Limited sample size; cross-sectional design; sequencing does not assess microbial viability
Malaluang et al., 2022; 2024 [44,50]	Vagina	Culture ± sequencing	Estrus/Diestrus	Frequent detection of *E. coli* and *S. zooepidemicus*; variation across cycle; culture highlights clinically relevant viable taxa	Culture bias toward fast-growing aerobes; limited detection of anaerobes; potential sampling and methodological variability
Castro-Chaves et al., 2013 [51]	Vagina	Culture + PCR	Estrus	Low *Lactobacillus* abundance; species-level differences compared with humans; targeted molecular confirmation	Small cohort; targeted detection limits broader community characterization; limited ecological interpretation
Fraga et al., 2008 [52]	Vagina	Culture	Estrus	Isolation of lactic acid bacteria with antimicrobial and biofilm-forming properties; potential probiotic relevance	Functional findings not linked to in vivo outcomes; lack of sequencing comparison; culture bias limits ecological scope
Hinrichs et al., 1988 [14]	Vagina/Uterus	Culture	Mixed	Viable bacteria were infrequently recovered from the uterus; findings support the concept of low uterine biomass and a caudal tract reservoir	The culture-only approach likely underestimates microbial diversity due to the inability to detect non-culturable or fastidious organisms
Thomson et al., 2022 [17]	Uterus	Sequencing	Estrus	Detection of bacterial DNA in clinically normal mares; *Proteobacteria* frequently dominant; supports non-sterile signal at DNA level	High susceptibility to contamination in low-biomass samples; limited assessment of viability; cross-sectional design
Heil et al., 2024 [45]	Uterus	Sequencing	Estrus/Anestrus	Relative stability at the phylum level with variation at the genus level across phases; suggests potential endocrine-associated trends	Cross-sectional design; limited functional validation; interpretation constrained by low biomass and lack of viability data
Holyoak et al., 2022 [16]	Uterus	Sequencing	Mixed	High inter-individual variability; proposed “core microbiome,” but composition is inconsistent across populations	Potential contamination influence; geographic variability; lack of longitudinal validation; core microbiome definition depends on analytical thresholds

**Table 4:** Comparative summary of stallion seminal microbiota according to methodological approaches.

Method	Dominant Phyla	Key Taxa Detected	Main Insights	Limitations
Culture-based studies	*Firmicutes*, *Proteobacteria*	*Staphylococcus*, *Micrococcus*, *Streptococcus*, *Escherichia coli*, *Pseudomonas*, *Klebsiella*	Detect viable, fast-growing aerobic or facultative microorganisms; reflect commensals of external genitalia and opportunistic pathogens relevant to clinical diagnostics	Strong bias toward fast-growing aerobes; underrepresentation of anaerobic and fastidious taxa; limited representation of total community diversity [61,62,63,64]
External genital microbiota (culture-based)	—	*Streptococcus dysgalactiae*, *Staphylococcus aureus*, *Escherichia coli*	External genitalia are a major source of microorganisms detected in semen, highlighting the importance of contamination during collection	There is a high risk contamination, making it difficult to distinguish endogenous seminal microbiota from external sources [62,63]
16S rRNA gene sequencing (NGS)	*Firmicutes*, *Bacteroidetes*, *Actinobacteria*, *Proteobacteria*	*Porphyromonas*, *Peptoniphilus*, *Corynebacterium*, *Prevotella*	Reveals broader microbial DNA diversity, including anaerobic and low-abundance taxa; suggests recurrent phylum-level patterns with high inter-individual variability	Detects DNA from viable and nonviable organisms; highly sensitive to contamination; limited functional resolution [32,33,34,37]
16S sequencing (population-level variation)	*Firmicutes*, *Bacteroidetes*	*Peptoniphilus*, *Prevotella*	Demonstrates geographic, environmental, and management-associated variation in microbial profiles	Confounded by environmental factors and sampling variability; limited standardization across studies [34,37]
16S sequencing (breed-specific datasets)	*Firmicutes*, *Bacteroidetes*	*Porphyromonas*, *Suttonella*, *Peptoniphilus*	Confirms recurrent phyla across breeds; highlights high individual variability and absence of a consistent core microbiota	Small sample sizes; limited generalizability; cross-sectional design [35]
Shotgun metagenomics	*Firmicutes*, *Proteobacteria*	*Staphylococcus*, *Escherichia*, *Pseudomonas*	Provides functional insights, including antimicrobial resistance genes and metabolic potential	Limited studies; higher cost; still unable to confirm microbial viability [65]
NGS (associations with semen quality)	*Firmicutes*, *Actinobacteria*	*Peptoniphilus*, *Clostridiales*-related taxa	Reports associations between specific taxa and sperm motility parameters; suggests potential microbiota–sperm interactions	Associations are correlative; causality is not established; findings are inconsistent across studies [32,33,34,37]
NGS (pathogen carriage status)	*Actinobacteria*, *Bacteroidetes*, *Firmicutes*	*Corynebacterium*, *Porphyromonas*, *Bacteroides*	Pathogen carriage, such as *Taylorella equigenitalis*, may be associated with altered microbial profiles	Limited longitudinal evidence; difficulty distinguishing pathogen effects from background variability [36]

## Data Availability

All the datasets in this study can be provided upon reasonable request.

## References

[1] Khan I.U., Khairullah A.R., Khan A.Y., Rehman A.U., Mustofa I. (2025). Strategic Approaches to Improve Equine Breeding and Stud Farm Outcomes. Vet. World.

[2] Palmer E., Chavatte-Palmer P. (2020). Contribution of Reproduction Management and Technologies to Genetic Progress in Horse Breeding. J. Equine Vet. Sci..

[3] Rambags B.P.B., Colenbrander B., Stout T.A.E. (2003). Early Pregnancy Loss in Aged Mares: Probable Causes and Possible Cures. Pferdeheilkunde.

[4] Ball B.A. (1988). Embryonic Loss in Mares: Incidence, Possible Causes, and Diagnostic Considerations. Vet. Clin. North Am. Equine Pract..

[5] Hurtgen J.P. (1992). Evaluation of the Stallion for Breeding Soundness. Vet. Clin. North Am. Equine Pract..

[6] Blanchard T.L., Thompson J.A., Brinsko S.P., Varner D.D., Love C.C., Ramsey J., O’Meara A. (2010). Some Factors Associated With Fertility of Thoroughbred Stallions. J. Equine Vet. Sci..

[7] Schrimpf R., Gottschalk M., Metzger J., Martinsson G., Sieme H., Distl O. (2016). Screening of Whole Genome Sequences Identified High-Impact Variants for Stallion Fertility. BMC Genom..

[8] Tanner J.C., Barrell G.K. (2024). Reproductive Performance of a Cohort of Standardbred Mares under a Commercial Breeding System. Equine Vet. J..

[9] Lawson J.M., Shilton C.A., Lindsay-McGee V., Psifidi A., Wathes D.C., Raudsepp T., De Mestre A.M. (2024). Does Inbreeding Contribute to Pregnancy Loss in Thoroughbred Horses?. Equine Vet. J..

[10] Roach J.M., Arango-Sabogal J.C., Smith K.C., Foote A.K., Verheyen K.L., de Mestre A.M. (2022). Multivariable Analysis to Determine Risk Factors Associated with Abortion in Mares. Reprod. Fertil..

[11] Wobbe M., Reinhardt F., Reents R., Tetens J., Stock K.F. (2022). Quantifying the Effect of Warmblood Fragile Foal Syndrome on Foaling Rates in the German Riding Horse Population. PLoS ONE.

[12] Morris L.H.A., McCue P.M., Aurich C. (2020). Equine Endometritis: A Review of Challenges and New Approaches. Reproduction.

[13] Ricketts S.W., Mackintosh M.E. (1987). Role of Anaerobic Bacteria in Equine Endometritis. J. Reprod. Fertil. Suppl..

[14] Hinrichs K., Cummings M.R., Sertich P.L., Kenney R.M. (1988). Clinical Significance of Aerobic Bacterial Flora of the Uterus, Vagina, Vestibule, and Clitoral Fossa of Clinically Normal Mares. J. Am. Vet. Med. Assoc..

[15] Gil-Miranda A., Macnicol J., Orellana-Guerrero D., Samper J.C., Gomez D.E. (2024). Reproductive Tract Microbiota of Mares. Vet. Sci..

[16] Holyoak G.R., Premathilake H.U., Lyman C.C., Sones J.L., Gunn A., Wieneke X., DeSilva U. (2022). The Healthy Equine Uterus Harbors a Distinct Core Microbiome plus a Rich and Diverse Microbiome That Varies with Geographical Location. Sci. Rep..

[17] Thomson P., Pareja J., Núñez A., Santibáñez R., Castro R. (2022). Characterization of Microbial Communities and Predicted Metabolic Pathways in the Uterus of Healthy Mares. Open Vet. J..

[18] Krekeler N., Legione A., Perriam W., Finan S., Heil B.A., Burden C.A., McKinnon A.O., Marth C.D. (2023). Association of the uterine microbiome to mare fertility. J. Equine Vet. Sci..

[19] Berg G., Rybakova D., Fischer D., Cernava T., Vergès M.-C.C., Charles T., Chen X., Cocolin L., Eversole K., Corral G.H. (2020). Microbiome Definition Re-Visited: Old Concepts and New Challenges. Microbiome.

[20] Poole R.K., Soffa D.R., McAnally B.E., Smith M.S., Hickman-Brown K.J., Stockland E.L. (2023). Reproductive Microbiomes in Domestic Livestock: Insights Utilizing 16S rRNA Gene Amplicon Community Sequencing. Animals.

[21] Glassing A., Dowd S.E., Galandiuk S., Davis B., Chiodini R.J. (2016). Inherent Bacterial DNA Contamination of Extraction and Sequencing Reagents May Affect Interpretation of Microbiota in Low Bacterial Biomass Samples. Gut Pathog..

[22] Salter S.J., Cox M.J., Turek E.M., Calus S.T., Cookson W.O., Moffatt M.F., Turner P., Parkhill J., Loman N.J., Walker A.W. (2014). Reagent and Laboratory Contamination Can Critically Impact Sequence-Based Microbiome Analyses. BMC Biol..

[23] Karstens L., Asquith M., Davin S., Fair D., Gregory W.T., Wolfe A.J., Braun J., McWeeney S. (2019). Controlling for Contaminants in Low-Biomass 16S rRNA Gene Sequencing Experiments. mSystems.

[24] Davis N.M., Proctor D.M., Holmes S.P., Relman D.A., Callahan B.J. (2018). Simple Statistical Identification and Removal of Contaminant Sequences in Marker-Gene and Metagenomics Data. Microbiome.

[25] Fierer N., Leung P.M., Lappan R., Eisenhofer R., Ricci F., Holland S.I., Dragone N., Blackall L.L., Dong X., Dorador C. (2025). Guidelines for Preventing and Reporting Contamination in Low-Biomass Microbiome Studies. Nat. Microbiol..

[26] Selway C.A., Eisenhofer R., Weyrich L.S. (2020). Microbiome Applications for Pathology: Challenges of Low Microbial Biomass Samples during Diagnostic Testing. J. Pathol. Clin. Res..

[27] Canisso I.F., Segabinazzi L.G.T.M., Fedorka C.E. (2020). Persistent Breeding-Induced Endometritis in Mares—A Multifaceted Challenge: From Clinical Aspects to Immunopathogenesis and Pathobiology. Int. J. Mol. Sci..

[28] Katila T., Ferreira-Dias G. (2022). Evolution of the Concepts of Endometrosis, Post Breeding Endometritis, and Susceptibility of Mares. Animals.

[29] Guo L., Holyoak G.R., DeSilva U. (2025). Endometrial Microbiome in Mares with and without Clinical Endometritis. Front. Vet. Sci..

[30] Petersen C., Round J.L. (2014). Defining Dysbiosis and Its Influence on Host Immunity and Disease. Cell. Microbiol..

[31] Wang N., Chen L., Yi K., Zhang B., Li C., Zhou X. (2022). The Effects of Microbiota on Reproductive Health: A Review. Crit. Rev. Food Sci. Nutr..

[32] Quiñones-Pérez C., Hidalgo M., Ortiz I., Crespo F., Vega-Pla J.L. (2021). Characterization of the Seminal Bacterial Microbiome of Healthy, Fertile Stallions Using next-Generation Sequencing. Anim. Reprod..

[33] Quiñones-Pérez C., Martínez A., Ortiz I., Crespo F., Vega-Pla J.L. (2022). The Semen Microbiome and Semen Parameters in Healthy Stallions. Animals.

[34] Malaluang P., Niazi A., Guo Y., Nagel C., Guimaraes T., Rocha A., Aurich C., Morrell J.M. (2024). Bacterial Diversity in Semen from Stallions in Three European Countries Evaluated by 16S Sequencing. Vet. Res. Commun..

[35] Cooke C.G., Gibb Z., Grupen C.G., Schemann K., Deshpande N., Harnett J.E. (2024). The Semen Microbiome of Miniature Pony Stallions. Reprod. Fertil. Dev..

[36] Quiñones-Pérez C., Martínez A., Crespo F., Vega-Pla J.L. (2020). Comparative Semen Microbiota Composition of a Stallion in a Taylorella Equigenitalis Carrier and Non-Carrier State. Animals.

[37] Núñez-Montero K., Leal K., Rojas-Villalta D., Castro M., Larronde C., Wagenknecht L., Contreras M.J. (2024). 16s Gene Metagenomic Characterization in Healthy Stallion Semen. Res. Vet. Sci..

[38] Morel M.C.G.D. (2008). Equine Reproductive Physiology, Breeding and Stud Management.

[39] Pinto C.R.F. (2017). Reproductive Evaluation of the Mare. Manual of Clinical Procedures in the Horse.

[40] Paccamonti D.L., Lyle S.K. (2010). A Clinical View of Mare Reproductive Physiology. Pferdeheilkunde Equine Med..

[41] McCue P.M. (2014). Reproductive Evaluation of the Mare. Equine Reproductive Procedures.

[42] Satué K., Gardon J.C. (2016). Infection and Infertility in Mares. Genital Infections and Infertility.

[43] Barba M., Martínez-Boví R., Quereda J.J., Mocé M.L., Plaza-Dávila M., Jiménez-Trigos E., Gómez-Martín Á., González-Torres P., Carbonetto B., García-Roselló E. (2020). Vaginal Microbiota Is Stable throughout the Estrous Cycle in Arabian Mares. Animals.

[44] Malaluang P., Åkerholm T., Nyman G., Lindahl J., Hansson I., Morrell J.M. (2024). Bacteria in the Healthy Equine Vagina during the Estrous Cycle. Theriogenology.

[45] Heil B.A., van Heule M., Thompson S.K., Kearns T.A., Beckers K.F., Oberhaus E.L., King G., Daels P., Dini P., Sones J.L. (2024). Metagenomic Characterization of the Equine Endometrial Microbiome during Anestrus. J. Equine Vet. Sci..

[46] Squires E.L., McKinnon A.O., Shideler R.K. (1988). Use of Ultrasonography in Reproductive Management of Mares. Theriogenology.

[47] Aurich C. (2011). Reproductive Cycles of Horses. Anim. Reprod. Sci..

[48] Honnens A., Weisser S., Welter H., Einspanier R., Bollwein H. (2011). Relationships Between Uterine Blood Flow, Peripheral Sex Steroids, Expression of Endometrial Estrogen Receptors and Nitric Oxide Synthases During the Estrous Cycle in Mares. J. Reprod. Dev..

[49] Watson E.D., Skolnik S.B., Zanecosky H.G. (1992). Progesterone and Estrogen Receptor Distribution in the Endometrium of the Mare. Theriogenology.

[50] Malaluang P., Wilén E., Frosth S., Lindahl J., Hansson I., Morrell J.M. (2022). Vaginal Bacteria in Mares and the Occurrence of Antimicrobial Resistance. Microorganisms.

[51] Castro-Chaves M.M.B., Borges A.S., Oliveira-Filho J.P., Brolazo E.M., Ramires-Neto C., Alvarenga M.A. (2013). A Comparison of Microbiological and Molecular Detection of Vaginal Lactobacillus Spp. between Mares and Women. Anim. Reprod..

[52] Fraga M., Perelmuter K., Delucchi L., Cidade E., Zunino P. (2008). Vaginal Lactic Acid Bacteria in the Mare: Evaluation of the Probiotic Potential of Native *Lactobacillus* Spp. and *Enterococcus* Spp. Strains. Antonie Leeuwenhoek.

[53] Donato G.G., Nebbia P., Stella M.C., Gionechetti F., Ala U., Cristofoli D., Robino P., Pallavicini A., Nervo T. (2026). The Uterine Microbiota in Mares With Endometritis: Impacts of Antibiotic Treatment. Vet. Med. Int..

[54] Donato G.G., Necchi D., Gionechetti F., Ala U., Nebbia P., Robino P., Stella M.C., Vandaele H., Pallavicini A., Nervo T. (2026). Characterization of the Endometrial Microbiota of Healthy Mares Across the Estrous Cycle. Animals.

[55] Tyrnenopoulou P., Fthenakis G.C. (2023). Clinical Aspects of Bacterial Distribution and Antibiotic Resistance in the Reproductive System of Equids. Antibiotics.

[56] Morrell J.M., Rocha A. (2022). A Novel Approach to Minimising Acute Equine Endometritis That May Help to Prevent the Development of the Chronic State. Front. Vet. Sci..

[57] Marchesi J.R., Ravel J. (2015). The Vocabulary of Microbiome Research: A Proposal. Microbiome.

[58] Beckers K.F., Liu C.-C., Gomes V.C.L., Schulz C.J., Childers G.W., Fedorka C.E., Sones J.L. (2025). Effects of Intra-Uterine Ceftiofur on the Equine Uterine Microbiome. Vet. Sci..

[59] Malaluang P., Wilén E., Frosth S., Lindahl J.F., Hansson I., Morrell J.M. (2023). Antimicrobial Resistance in Vaginal Bacteria in Inseminated Mares. Pathogens.

[60] Amann R.P. (1981). A Review of Anatomy and Physiology of the Stallion. J. Equine Vet. Sci..

[61] Samper J.C. (2008). Equine Breeding Management and Artificial Insemination.

[62] Maasen M., Christensen P. (1995). Bacterial Flora of Semen Collected from Danish Warmblood Stallions by Artificial Vagina. Acta Vet. Scand..

[63] Pasing S.S., Aurich C., von Lewinski M., Wulf M., Krüger M., Aurich J.E. (2013). Development of the Genital Microflora in Stallions Used for Artificial Insemination throughout the Breeding Season. Anim. Reprod. Sci..

[64] Al-Kass Z., Eriksson E., Bagge E., Wallgren M., Morrell J.M. (2019). Bacteria Detected in the Genital Tract, Semen or Pre-Ejaculatory Fluid of Swedish Stallions from 2007 to 2017. Acta Vet. Scand..

[65] Al-Kass Z., Eriksson E., Bagge E., Wallgren M., Morrell J.M. (2020). Microbiota of Semen from Stallions in Sweden Identified by MALDI-TOF. Vet. Anim. Sci..

[66] Al-Kass Z., Guo Y., Vinnere Pettersson O., Niazi A., Morrell J.M. (2020). Metagenomic Analysis of Bacteria in Stallion Semen. Anim. Reprod. Sci..

[67] Rasmussen C.D., Haugaard M.M., Petersen M.R., Nielsen J.M., Pedersen H.G., Bojesen A.M. (2013). Streptococcus Equi Subsp. Zooepidemicus Isolates from Equine Infectious Endometritis Belong to a Distinct Genetic Group. Vet. Res..

[68] Wilson M., Williams J., Montrose V.T., Williams J. (2019). Variance in Stallion Semen Quality among Equestrian Sporting Disciplines and Competition Levels. Animals.

[69] Jlassi M., Jemmali B., Ouzari H.I., Lasfer F., Aoun B.B., Ben Gara A. (2023). Reproductive Performance of Tunisian Arabian Stallions: A Study on the Variance and Estimation of Heritability. Animals.

[70] Hernández-Avilés C., Zambrano-Varón J., Jiménez-Escobar C. (2019). Current Trends on Stallion Semen Evaluation: What Other Methods Can Be Used to Improve Our Capacity for Semen Quality Assessment?. J. Vet. Androl..

[71] Egyptien S., Deleuze S., Ledeck J., Ponthier J. (2023). Sperm Quality Assessment in Stallions: How to Choose Relevant Assays to Answer Clinical Questions. Animals.

[72] Khan I.M., Nassar N., Chang H., Khan S., Cheng M., Wang Z., Xiang X. (2024). The Microbiota: A Key Regulator of Health, Productivity, and Reproductive Success in Mammals. Front. Microbiol..

[73] Bhattacharya K., Dutta S., Sengupta P., Bagchi S. (2023). Reproductive Tract Microbiome and Therapeutics of Infertility. Middle East Fertil. Soc. J..

[74] Arıkan M., Muth T. (2023). Integrated Multi-Omics Analyses of Microbial Communities: A Review of the Current State and Future Directions. Mol. Omics.

[75] Sabih Ur Rehman S., Nasar M.I., Mesquita C.S., Al Khodor S., Notebaart R.A., Ott S., Mundra S., Arasardanam R.P., Muhammad K., Alam M.T. (2025). Integrative Systems Biology Approaches for Analyzing Microbiome Dysbiosis and Species Interactions. Brief. Bioinform..

[76] Singh B., Bhat A., Ravi K. (2024). Antibiotics Misuse and Antimicrobial Resistance Development in Agriculture: A Global Challenge. Environ. Health.

[77] Zabala S.M., Serres C., Montero N., Crespo F., Lorenzo P.L., Pérez-Aguilera V., Oliet A., Hijón V., Moreno S., González-Zorn B. (2025). Innovative Approaches to Avoid Antibiotic Use in Equine Semen Cryopreservation: Advancing Sustainable Reproductive Technologies. Animals.

